# The Cost-Effectiveness and Value of Information of Three Influenza Vaccination Dosing Strategies for Individuals with Human Immunodeficiency Virus

**DOI:** 10.1371/journal.pone.0027059

**Published:** 2011-12-06

**Authors:** Bohdan Nosyk, Behnam Sharif, Huiying Sun, Curtis Cooper, Aslam H. Anis, on behalf of the CIHR Canadian HIV Trials Network Influenza Vaccine Research Group

**Affiliations:** 1 CIHR Canadian HIV Trials Network, Vancouver, British Columbia, Canada; 2 Division of Infectious Diseases, University of Ottawa, Ottawa, Canada; 3 School of Population and Public Health, University of British Columbia, Vancouver, British Columbia, Canada; Statens Serum Institute, Denmark

## Abstract

**Background:**

Influenza vaccine immunogenicity is diminished in patients living with HIV/AIDS. We evaluated the cost-effectiveness and expected value of perfect information (EVPI) of three alternative influenza vaccine dosing strategies intended to increase immunogenicity in those patients.

**Methods:**

A randomized, multi-centered, controlled, vaccine trial was conducted at 12 CIHR Canadian HIV Trials Network sites. Three dosing strategies with seasonal, inactivated trivalent, non-adjuvanted intramuscular vaccine were used in HIV infected adults: two standard doses over 28 days (Strategy A), two double doses over 28 days (Strategy B) and a single standard dose of influenza vaccine (Strategy C), administered prior to the 2008 influenza season. The comparator in our analysis was practice in the previous year, in which 82.8% of HIV/AIDS received standard-dose vaccination (Strategy D). A Markov cohort model was developed to estimate the monthly probability of Influenza-like Illness (ILI) over one influenza season. Costs and quality-adjusted life years, extrapolated to the lifetime of the hypothetical study cohorts, were estimated in calculating incremental cost-effectiveness ratios (ICER) and EVPI in conducting further research.

**Results:**

298 patients with median CD4 of 470 cells/µl and 76% with viral load suppression were randomized. Strategy C was the most cost-effective strategy for the overall trial population and for suppressed and unsuppressed individuals. Mean ICERs for Strategy A for unsuppressed patients could also be considered cost-effective. The level of uncertainty regarding the decision to implement strategy A versus C for unsuppressed individuals was high. The maximum acceptable cost of reducing decision uncertainty in implementing strategy A for individuals with unsuppressed pVL was $418,000 - below the cost of conducting a larger-scale trial.

**Conclusion:**

Our results do not support a policy to implement increased antigen dose or booster dosing strategies with seasonal, inactivated trivalent, non-adjuvanted intramuscular vaccine for individuals with HIV in Canada.

**Trial Registration:**

ClinicalTrials.gov NCT00764998.

## Introduction

The likelihood of being clinically protected after influenza vaccination is diminished in those living with HIV/AIDS [1]. Influenza symptoms are prolonged and the risks of complications resulting from influenza infection are increased [2]. Furthermore, the risk of influenza-related mortality is increased [2], [3].

Current guidelines recommend that individuals with HIV/AIDS receive the same standard influenza vaccination dosing strategy as the general population (i.e. a single standard dose of inactivated influenza vaccine administered annually in October/November) [2], [4]. This is supported by several studies and meta-analyses suggesting reduced risk of influenza cases [5], [6], [7]. Higher vaccine doses and/or booster dosing may maximize seroconversion and seroprotection in individuals with HIV [1], [8]. However, few randomized studies of alternative dosing strategies have been conducted to determine the optimal approach to achieving this goal [1]. A recent randomized trial conducted in Canada during the 2008 influenza season found that even with increased antigen dose and booster dosing, non-adjuvanted influenza vaccine immunogenicity is poor in HIV-infected individuals [9]. However, there was a great deal of uncertainty regarding the level of clinical protection between dosing strategies, and within different patient strata. As such, it is of interest to determine the cost-effectiveness of the different strategies and to determine whether further research to reduce the uncertainty in the decision to implement these strategies is cost-effective.

We conducted a cost-effectiveness analysis utilizing data generated from a randomized trial to determine the incremental cost per quality-adjusted life year and expected value of perfect information of conducting further research for three different vaccine dosing strategies for people with HIV/AIDS.

## Methods

### Patient Population and Interventions

A randomized controlled trial assessing the immunogenicity and efficacy of three influenza vaccine dosing strategies among individuals with HIV was conducted in Canada during the 2008–2009 influenza season (CTN-237). The vaccine used was the 2008 seasonal trivalent killed split non-adjuvanted influenza vaccine (FluviralTM, GSK, Laval, Canada) containing A/Brisbane/59/2007 (H1N1), A/Uruguay/716/2007 (H3N2), and B/Florida/4/2006. The trial allocated patients randomly to three influenza dosing strategies:

1. Strategy A (single standard dose+single standard dose booster): 15 mg dose of Fluarix ® influenza vaccine on Day 0 and Day 28.
2. Strategy B (double dose+double dose booster): 30 mg dose of Fluarix ® influenza vaccine on Day 0 and Day 28.
3. Strategy C: (single standard dose+no booster): 15 mg dose of Fluarix ® influenza vaccine on Day 0 and placebo on Day 28.

Randomization was stratified by CD4 T lymphocyte count (<200 cells/mL versus ≥200 cells/mL). Participants and all study staff were blinded to allocation, except for the individual who prepared the vaccine who had no direct contact with study participants.

Hemagglutination inhibition (HAI) titres were measured at Laval University (Dr Guy Boivin) according to WHO standard protocol. Briefly, non-specific inhibitors were removed from serum by overnight treatment with receptor destroying enzyme (Denka Seiken, Tokyo, Japan). Physiologic saline solution was then added to achieve a 1∶10 dilution, followed by incubation with packed guinea pig red blood cells (GRBC) (Lampire Biological Laboratories Inc., Pipersville, PA) at 4 uC for 60 min to remove non-specific agglutinins. Treated serum was serially diluted in 25 ml of PBS and then mixed with an equal volume of PBS containing 4 hemagglutinin units of A/Brisbane/59/2007 (H1N1), A/Uruguay/716/2007 (H3N2) or B/Florida/4/2006 viruses. After 30 min of incubation at room temperature, 50 ml of 1% GRBC solution was added to the mixture and incubated for 45–60 min before evaluation of hemagglutination. The HAI titer was recorded as the reciprocal of the last dilution that inhibited hemagglutination. These were measured at baseline, week 4, week 8 and week 20. Further details on serological testing and trial procedures can be found in Cooper et al. [9].

### Decision Analytic Model

This study was conducted from a societal perspective. Given that influenza vaccination is required on an annual basis to maintain protection against three different strains that mutate over time (i.e. genetic drift) we considered a one-year vaccination period, with costs and health-related quality of life outcomes extrapolated beyond the one-year period to the lifetime of the hypothetical cohorts.

We developed a Markov cohort model to track disease progression through states of HIV plasma virological suppression (≤50 copies per ml), non-suppression (>50 copies per ml) and death over one full influenza season. Each hypothetical cohort had an equal mix of individuals who were virologically suppressed, according to baseline trial figures. Costs and Health-related quality of life outcomes of survivors were extrapolated beyond this 12-month period for the estimated life expectancy of HIV/AIDS patients at age 35 and residing in high-income countries [10]. Costs and QALYs were discounted at a rate of 3% per annum, according to national and international guidelines for cost-effectiveness analysis [11].

**Figure 1:**
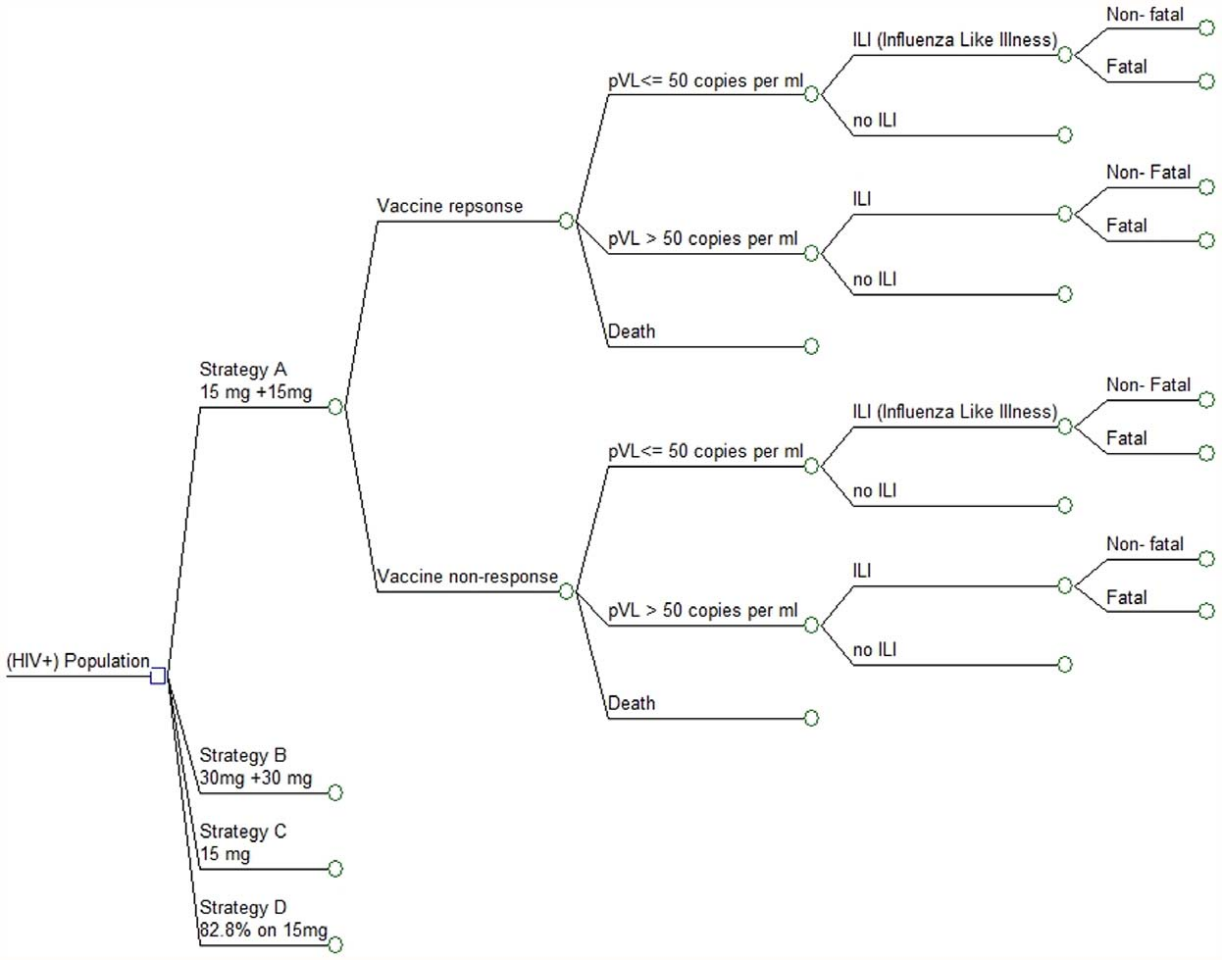
Decision analytic model. All nodes following vaccine response are repeated each month throughout the initial 12 months of the model duration; therefore patients not suffering fatal ILI or death due to other causes may transition from HIV viral load suppression to non-suppression, and subsequently face differential risk of ILI at each month. The probability of ILI is summed across each of the three strains of influenza assessed in CTN-237.

Transition probabilities between states were estimated from a systematic review [12], the UK Collaborative HIV Cohort Study [13], and the Antiretroviral Therapy Cohort Collaboration study [10]. Using trial-based data, HAI titre levels were estimated for each health state and each dosing strategy over the one-year time horizon. We then estimated the probability of ILI in each health state, using a mathematical relationship to solve for the rate of clinical protection at each HAI titre level, and subsequently, the probability of ILI. The decision-analytic model is illustrated in Figure 1, while additional parameters required for the model are presented in Table 1.

**Table 1:** Model parameters.

	Estimate		Source
*HIV disease progression*			
Probability of transition: HIV plasma viral load suppressed to unsuppressed*	80.7/1000PY		[13]
Probability of transition: HIV plasma viral load unsuppressed to suppressed#	68/131		[12]
Probability of transition: mortality:			[10]
CD4<25	38.4/1000PY		
CD4 25–49	29.5/1000PY		
CD4 50–99	26.4/1000PY		
CD4 100–199	18.7/1000PY		
CD4 200–349	10.9/1000PY		
CD4≥350	6.9/1000PY		
Life Expectancy - years	32 (0.21)		[10]
Annual HIV healthcare cost			
CD4≤50	$40,678 (95% CI: 33,566–47,789)		[29]
CD4 51–200	$26,011 (95% CI: 23730,28292)		
CD4 201–350	$19,565 (95% CI: 18,472, 20,658)		
CD4 351–500	$16,859 (95% CI: 15,798, 17,920)		
CD4>500	$16,614 (95% CI: 16,052 ,17,177)		
*Influenza-like Illness*			
ILI Attack rate: proportion of patients with ILI within 1 year	182/1000PY		[20]
Vaccine coverage: General population	32%		[14]
Vaccine coverage: HIV+ population [%]	241/291 [82.8%]		CTN-237
Estimated HAI titre in general population**:			[22]
Influenza Strain	**A H1N1**	**A H3N2**	**B**
Week 0	15.0 (15.0)	22.4 (32.1)	52.5 (71.8)
Week 4	156.2 135.0)	324.3 (348.0)	232.6(127.0)
Week 20	50.3 (33.1)	84.6 (39.0)	64.0 (33.7)
Probability of mortality due to ILI event	9.9/1000 ILI cases		[25]
HRQoL: Non-symptomatic patients***	0.835 (0.01)		CTN-237
HRQoL loss due to ILI	0.002		CTN-237
Estimated cost per ILI case##	$672.76 (95% CI: 358.62, 1037.07)		CTN-237, [30]

ILI: Influenza like illness; PY: Person-year; HRQoL: Health-related quality of life;

*Among pre-treated patients.

**Drawn from 2000/2001 estimates among a healthy elderly population [20].

# Unsuppressed patients were treated with one new drug; percentage of patients transitioning within a one-year period.

***Derived from baseline HUI3 scores of CTN-237 participants.

## In 2009$CDN. Included the costs of Derived from 5000 bootstrap re-samples of N = 31 ILI events captured in the CTN-237 database.

We calculated the incremental costs per quality-adjusted life year gained for each of the three dosing strategies in comparison to current practice, in which vaccine coverage was not complete. 82.8% of subjects recruited for this study reported being vaccinated in the prior year. The comparator cohort, Strategy D, for this study was therefore constructed from results of strategy C (assumed standard single-dose vaccination) for 82.8% of the cohort; estimates of mean HAI titre for patients in Strategy D were calculated by weighting the mean HAI titre of patients in Strategy C and HAI titre = 10; the assumed HAI titre for non-vaccination patients. The coverage percentage for general population has been derived from the 2007 Canadian Community Health Survey [14].

#### Estimating the probability of ILI

Previously, Dunning et al. [15] proposed a scaled logistic model to estimate clinical protection as a function of HAI titre. Nauta et al. [16] applied a similar equation to determine the expectation of clinical protection and demonstrated that the variance of HAI titre is also an important determinant of clinical protection. We have expanded this model to estimate the probability of contracting influenza for each month by adding the exposure parameters for three strains of the influenza virus.

The monthly probability of influenza for each strain (H1N1, H3N2, or B) for our study population was a function of the HAI titre level, which provides the expectation of clinical protection to the influenza virus, and the probability that an individual with HIV who was not clinically protected developing ILI due to the virus. We described the methods used to calculate each component below.

#### Clinical protection

For a given individual with HAI titre measured at a given month, *t = log_2_ (HAI titre/10)* shows the log transformed HAI titre. The clinical protection function is given by *π (t,α,β)*, which gives the relationship between *t* and the probability of clinical protection from influenza.
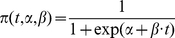
(1)


For a given rate of clinical protection at *t = 0* and threshold at which clinical protection is equal to 50%, *α* and β can be derived from Equation 1. We assumed *α = 6*, which gave a protection rate 0.0025 for *t = 0*, and an HAI titre threshold = 40 as our base case [17], [18]. We also assumed that for a given group of individuals and a given strain, *t* follows a normal distribution with mean *μ* and standard deviation *σ*, consistent with prior studies [15], [16]. We then estimated the expectation of the probability of clinical protection for a given population by:
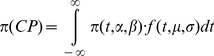
(2)


Where *f(t, μ, σ)* is the normal density function of *t* with mean *μ* and standard deviation *σ*, and *π*(CP) is the mean fraction of subjects who are actually clinically protected [16]. It reflects the clinical protection at the time of exposure to influenza virus among those individuals with the mean of *t* equal to *μ* and the standard deviation of *t* equal to σ. The integrals were solved numerically using MATLAB 7.1 statistical toolbox.

#### Probability of contracting ILI

For each dosing strategy in a given month, the mean probability of a population with given distribution of HAI titre contracting a particular strain of influenza can be expressed as follows:
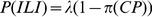
(3)


In Equation 3, *λ* is the probability that a susceptible individual develops influenza. *π*(CP) is calculated from the data on HAI titre of each group of individuals in our study as discussed above in section 1 (Clinical protection). However, as we did not have any data on λ for our study population, we assumed that λ is the same for both general and HIV/AIDS population. The probability of ILI was therefore calculated as:
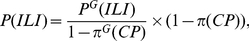
(4)


Where P^G^(ILI) is the probability of contracting ILI for general population and π^G^(CP) is the mean fraction of subjects who are clinically protected among the general population. P^G^(ILI) at each month is known by utilizing annual attack rate estimates from peer-reviewed literature on the monthly distribution of ILI cases form surveillance data [19]. *π^G^(CP)* was derived from the literature [20], [21], [22] and *π (CP)* is a function of HAI titre of our study population and can be calculated from Equation 2. As a result, P(ILI) for each strain can be calculated form Equation 4. The mean probability of individuals with HIV/AIDS developing any of the three types of influenza is then calculated as the sum of strain-specific ILI probabilities at each month.

**Figure 2:**
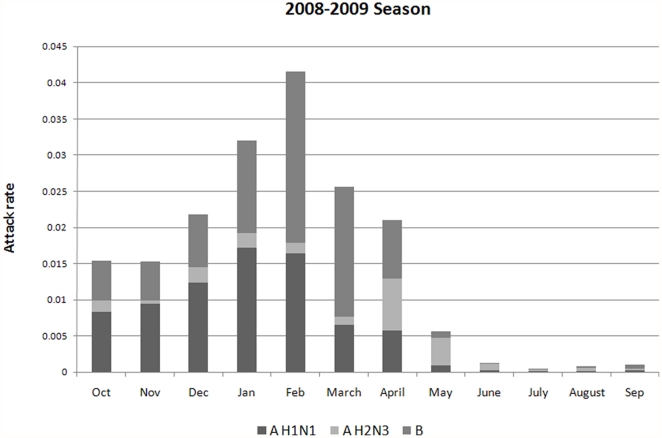
Monthly distribution of the probability of ILI. Weekly influenza surveillance report form CDC [22]. 2008–2009 influenza season, week 39 ending October 3, 2009. Data shows only seasonal influenza and pandemic strain, 2009 influenza A (H1N1) virus, has been omitted.

Literature estimates and surveillance data from the US Centre for Disease Control from 2002–2009 were used to estimate the monthly attack rate of ILI for each strain in the following manner: First, annual attack rates were derived from the literature [20], [21]. Sentinel data on the percentage of physician visits due to ILI was then used to derive the monthly distribution of the annual influenza attack rate [19] (Figure 2). Finally, US virologic surveillance data from lab reports was used to estimate the contribution of each three strains (H1N1, H3N2, and B) to the total ILI cases in each month [19]. We implicitly assumed that other influenza strains contributed a negligible number of ILI cases.

Thus, using estimates of the annual influenza attack rate for the general population [18], [19], the monthly probability of ILI over a typical influenza season [22], [23] and seasonal distributions of the particular strain of influenza [19], strain-and time-specific probability of ILI were estimated. The overall probability of ILI was then calculated as the sum of strain-specific ILI probabilities for each month.

#### Estimating Mean HAI Titre Levels

In order to calculate estimated HAI titre levels for each strain, dosing strategy and health state, we analyzed trial data on HAI trajectories and determinants of HAI titre improvement through the 20-week trial follow-up. As many trial participants did not show increases in HAI titre levels, we estimated a hurdle model with the probability of HAI titre improvement as the outcome in the first stage; and t = log_2_ (HAI titre/10) the outcome among only those with improvement in the second stage. HAI titre improvement was defined as HAI titre ever being greater than 1∶10 during follow-up. This definition was data-driven, and defined as such to enable us to make the most accurate predictions of ILI as a function of HAI titre level. Covariates included virological suppression (≤50 copies per ml), time, and dosing strategy interactions.

Thus, for each strategy and health state, we estimated the percentage of those with no HAI titre improvement and the annual titre trajectory among those with HAI titre improvement. For those with no improvement, t was assumed to remain equal to zero throughout the year. Among those with improvement, mean t and standard deviation at month 0 (weeks 0), month 1 (week 4), month 2 (week 8), and month 5 (week 20) were estimated from the trial data. The mean t for each strategy and health state was then extrapolated to months 3 and 4 by assuming a linear trend in mean titre between months 2 and 5, and extrapolated beyond the 5-month time horizon of the trial by assuming a linear trend in mean t decline to zero at the end of the 12-month period. The standard deviation at months 3 and 4, and beyond month 5 were extrapolated by max (0.5, k*mean), where k = average (σ_0_/μ_0_, σ_1_/μ_1_, σ_2_/μ_2_, σ_5_/μ_5_), μ_0_, μ_1_, μ_2_, μ_5_ and σ_0_, σ_1_, σ_2_, σ_5_ are the mean of t and standard deviation at months 0, 1, 2, and 5, respectively. The same methodology was applied to extrapolate HAI titre levels for the general population for which HAI titre data was available at weeks 0, 4 and 20 [22].

#### Estimating the probability of mortality due to influenza

We assumed that the influenza case-fatality rate of individuals with HIV/AIDS was equal to that of general population [24]. An estimate of the probability of ILI-related mortality for the general population was derived from the literature [25].

#### Health-related quality of life

The health utilities index (HUI-III), a valid and responsive measure of HRQoL for patients with HIV/AIDS [26], was collected prospectively among patients enrolled in this study at regular follow-up intervals. Additional assessments were made during ILI events, and the duration symptomatic ILI was reported to capture QALY loss due to ILI events.

The temporary decline in health-related quality of life due to ILI was estimated from the literature [27] given the low number of ILI cases in our trial. Patients lost an average of 0.002 QALYs due to ILI. QALYs were calculated for the duration of estimated life expectancy at the baseline level of HRQoL observed in the trial.

Health-related quality of life outcomes were then extrapolated to the lifetime of the patient cohort based on external estimates of life expectancy for HIV/AIDS patients of similar clinical prognosis [10].

#### Estimating the costs of ILI

Costs of influenza vaccines and ILI, including inpatient care, outpatient care and foregone productivity, were estimated from trial data. The human capital approach was used to estimate costs of foregone productivity among employed trial participants [28]. Costs were presented in 2009$CDN.

The lifetime costs of individuals with HIV/AIDS, sourced from external estimates [29], were then applied to the surviving cohort and discounted accordingly. Database analysis was conducted using SAS version 9.2, while the probabilistic cohort simulation model was constructed in Microsoft Excel.

### Incremental Cost-Effectiveness Analysis

Incremental cost-effectiveness ratios (ICERs) were calculated as the ratio of the difference in costs and the difference in the quality adjusted life years (QALYs) gained between each of the tested dosing strategies i and the current standard of care:
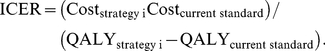
(5)


### Sensitivity Analysis

The deterministic results of our mathematical model were subject to first- and second-order uncertainty, due to variability in parameters, and the model structure [30]. Probabilistic sensitivity analysis was conducted to identify the uncertainty around ICERs and the subsequent decision rules generated from the analysis.

Uncertainty around parameter estimates based on trial data was quantified using non-parametric bootstrapping (5000 bootstrap re-samples), while Monte Carlo simulation (5000 simulated values) was used for parameters derived from external sources, in which only measures of central tendency and variation were available. 95% credibility intervals (CI) were presented for each estimated ICER.

Cost-effectiveness acceptability curves (CEACs), displaying the probability that a given intervention is more cost-effective than the alternative for a range of hypothetical maximum thresholds regarding society's willingness to pay for a single QALY.

Finally, one-way sensitivity analysis was conducted to determine the sensitivity of our results to changes in specific model parameters. In particular, we calculated ICERs (and 95% CI) for model formulations in which the age at model entry was set at 25 and 45 years, and extreme values of the annual attack rate and influenza case fatality rate.

### Value of Information Analysis

Uncertainty surrounding the mean estimate of cost-effectiveness can be costly if it increases the possibility of making the wrong decision in terms of implementing or not implementing the health intervention. The information gained from further research is valuable, as it reduces the expected costs of this uncertainty [31]. The Expected Value of Perfect Information (EVPI) can be interpreted as the expected costs of uncertainty, as perfect information would eliminate completely the possibility of making the wrong decision.

The EVPI is thus calculated as the difference between the expected value of benefit of which the decision would be made when the “true” parameter values were known (in other words, with ‘perfect’ information), and the expected value of benefit of which the decision would be made with uncertainty in all parameter values (current information), as follows:
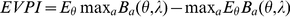
(6)


Where B_a_ represents the net benefit of a given strategy “*a*” (in monetary terms), which is a function of θ, the uncertain parameters, and λ, the threshold cost-effectiveness ratio.

The EVPI represents the maximum sum that the health care system should be willing to pay for reducing the uncertainty in the decision to implement the vaccine dosing strategy in question. Although the estimated EVPI is the expected maximum value for additional information to inform the treatment of a single patient, the information acquired can be used to treat all patients that may benefit from the intervention in question. We can therefore estimate the population, or total EVPI by multiplying the per-person EVPI by the number of persons that may benefit from intervention in a given year.

## Results

Characteristics of the 298 patients recruited for this study were presented in Table 2. Ninety percent were on HAART and 76% had HIV plasma viral load levels below detection (≤50 copies per ml) at baseline (strategy A: 79%; strategy B: 72%; strategy C: 77%; p = 0.30). The median CD4 count was 470 cells/ml. 47% of these patients were employed at the baseline assessment; the majority of which employed full-time.

**Table 2:** Patient characteristics.

	N (%)/Mean (SD)	
Age [mean (SD)]	46.8 (8.5)	
Female [N (%)]	29 (9.7)	
Ethnicity [N (%)]:		
Caucasian	241 (80.9)	
Black	21 (7.1)	
Other	36 (12.1)	
Employment [N (%)]:		
Full-time	109 (36.6)	
Part-time	31 (10.4)	
Not employed	158 (53.0)	
Virologically suppressed [pVL≤50 copies/ml] [N (%)]	227 (76.2)	
CD4 cell count [mean (SD)]:	Suppressed	Unsuppressed
CD4 25–49	0 (0.0)	1 (1.4)
CD4 50–99	1 (0.4)	3 (4.2)
CD4 100–199	17 (7.5)	8 (11.3)
CD4 200–349	40 (17.6)	19 (26.8)
CD4≥350	169 (74.5)	40 (56.3)

pVL = HIV plasma viral load.

**Figure 3:**
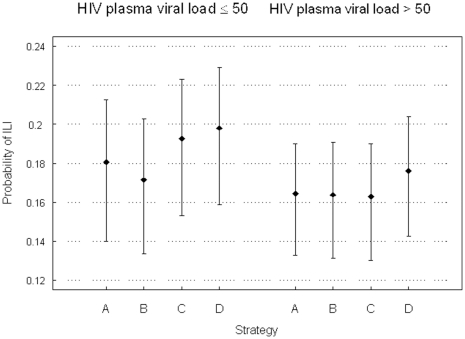
Mean of the probabilities of ILI and 95% credibility interval for each strategy by baseline pVL. Strategy A: single standard dose+single standard dose booster; Strategy B: double dose+double dose booster; Strategy C: single standard dose+no booster; Strategy D: standard of care.

Trial-based estimates of the probability of HAI titre response and estimated probability of ILI for each dosing strategy were presented in Table 3 and Figure 3, respectively. The probability of HAI titre response ranged from 0.55 (strategy A, unsuppressed, H3N2) and 0.86 (Strategy C, suppressed, H3N2), and was higher among patients with suppressed viral loads in all but 2 of 18 estimates. The estimated probabilities ILI among virologically suppressed patients were similar in treatment strategies A through C for each strain Figure 3. Among non-suppressed patients, treatment strategies B (double dose+double booster) and A (single standard dose+single standard dose booster) had lower probabilities of ILI than strategy C (single standard dose+no booster).

**Table 3:** Probability of HAI titre improvement*: results from 1^st^-stage analysis.

	Strategy A: single standard dose+single standard dose booster N (%)	Strategy B: double dose+double dose booster N (%)	Strategy C: single standard dose+no booster N (%)
*A H1N1 [Brisbane]*			
Baseline pVL≤50 copies/ml	52 (71.23)	55 (74.32)	53 (76.81)
Baseline pVL>50 copies/ml	14 (63.64)	20 (76.92)	13 (65.00)
*A H3N2 [Uruguay]*			
Baseline pVL≤50 copies/ml	58 (79.45)	58 (78.38)	59 (85.51)
Baseline pVL>50 copies/ml	12 (54.55)	18 (69.23)	11 (55.00)
*B [Florida]*			
Baseline pVL≤50 copies/ml	54 (73.97)	55 (74.32)	52 (75.36)
Baseline pVL>50 copies/ml	14 (63.64)	22 (84.62)	12 (60.00)

*HAI Titre improvement was defined as HAI titre ever being greater than 1∶10 during follow-up assessments.

The cost of a single administration of influenza vaccine was $17.45 ($8.25 for 15 mg of Fluarix [9], [32] plus $9.20 administrative cost [33]). Trial-based data was used to construct the costs of ILI events. There were 31 distinct ILI events reported among 28 patients. While hospitalization was rare (N = 1 (3.2%)), the majority of patients sought outpatient care (71.0%), and missed at least one day of work (61.3%). The estimated cost per ILI case was $672.76 (95% CI: $358.62, $1,037.07). Costs of hospital admission and inpatient care were derived from the St. Paul's Hospital Cost Model, which provides fully-allocated costs of all activities in an urban Canadian tertiary care teaching hospital [34].

Estimated costs, QALYs and ICERs for each dosing strategy in comparison to strategy D (standard of care) in the baseline model formulation were presented in Table 4. Strategy C (single standard dose+no booster) dominated (lower costs, higher QALYs) strategy D for the overall trial population. The ICER for strategy A (single standard dose+single standard dose booster) was $104,781 ($17,973, $2,939,656) per QALY gained in the overall trial population, $68,190 ($132, $2,085,500) for unsuppressed patients, and $122,152 ($19,307, Dominated) for virologically suppressed patients. Mean ICERs for strategy B were well above commonly used thresholds for the overall trial population, virologically suppressed patients and unsuppressed patients. Credibility intervals were generally wide due to the small differences in discounted lifetime QALYs gained between strategies, which were in the order of 0.001.

**Table 4:** Incremental costs per quality-adjusted life year gained: strategies A vs. Standard of Care.

		ICER vs. Standard of care [2009$CDN]		
		Strategy A:	Strategy B:	Strategy C:
	Standard of Care	doses: 1+1	doses: 2+2	doses: 1+0
*Trial cohort*				
Cost	$412,201 ($393,647, $425,977)	$412,215 ($393,665, $425,978)	$412,249 ($393,701, $426,010)	$412,198 ($393,645, $425,967)
QALY	17.72621 (16.99317, 18.26332)	17.72635 (16.99329, 18.26349)	17.72638 (16.99329, 18.26365)	17.72633 (16.99329, 18.26344)
ICER	–	$104,781 ($17,973, $2,939,656)	$291,656 ($120,986, $2,211,232)	D (D, $11,150)
*Suppressed pVL*				
Cost	$405,585 ($387,555, $419,332)	$405,599 ($387,566, $419,344)	$405,634 ($387,602, $419,384)	$405,581 ($387,550, $419,322)
QALY	17.72818 (16.99568, 18.25104)	17.72830 (16.99581, 18.25110)	17.72831 (16.99585, 18.25112)	17.72832 (16.99580, 18.25111)
ICER	–	$122,152 (19,307, DT)	$389,454 ($131,897, DT)	D (D, $7,644)
*Unsuppressed pVL*				
Cost	$433,506.00 ($402,319,$462,553)	$433,518.28 ($402,327, $462,557)	$433,549.15 ($402,363, $462,586)	$433,506.15 ($402,319,$462,552)
QALY	17.68808 (16.93064,18.21694)	17.68826 (16.93087,18.21716)	17.68836 (16.93087,18.21717)	17.68814 (16.93067,18.21610)
ICER	–	$68,190 ($132, $2,085,500)	$156,609 ($63,922, $704,783)	$2,722 (D, $63,943)

ICER = (Cost_strategy i_−Cost_current standard_)/(QALY_strategy i_−QALY_current standard_); D: Dominant - Lower cost, higher QALYs in comparison to usual care; DT: Dominated - higher cost, lower QALYs in comparison to usual care. * Threshold for attaining 50% clinical protection for HIV/AIDS patients.

Sensitivity analyses on several key model inputs were presented in Table 5. ICERs were larger with lower ILI attack rates, and more favourable with higher ILI attack rates. Results were relatively insensitive to changes in the age of model entry. The parameters for which we had no source specific to HIV/AIDS clients were the probability of developing ILI among susceptible individuals and the ILI case fatality rate. The estimate we've used was derived from a general population sample, and thus may underestimate the actual influenza case fatality rate for HIV/AIDS clients. A higher ILI case fatality rate led to more favourable ICERs indicating that Strategy A may be cost-effective for the trial cohort, including individuals with suppressed plasma viral load.

**Table 5:** Results of one-way sensitivity analyses.

	ICER vs. Strategy D: standard of care [2009$CDN]		
Model Formulation	Strategy A:	Strategy B:	Strategy C:
	doses: 1+1	doses: 2+2	doses: 1+0
*HAI Titre threshold* * * = 80*			
Trial cohort	$262,385	$534,570	$1,582
Suppressed pVL	$327,568	$702,927	Dominant
Unsuppressed pVL	$153,842	$294,590	$61,258
*Influenza Attack Rate = 10%*			
Trial cohort	$231,392	$530,720	Dominant
Suppressed pVL	$271,824	$702,238	Dominant
Unsuppressed pVL	$152,685	$289,603	$31,936
*Influenza Attack Rate = 50%*			
Trial cohort	$11,105	$71,064	Dominant
Suppressed pVL	$18,872	$105,045	Dominant
Unsuppressed pVL	Dominant	$23,798	Dominant
*Age of model entry = 25*			
Trial cohort	$99,288	$248,109	Dominant
Suppressed pVL	$119,168	$333,112	Dominant
Unsuppressed pVL	$60,803	$128,905	$803
*Age of model entry = 45*			
Trial cohort	$122,278	$315,955	Dominant
Suppressed pVL	$148,324	$426,855	Dominant
Unsuppressed pVL	$71,744	$160,335	Dominant
*Influenza Case Fatality Rate = 0.1%*			
Trial cohort	$70,733	$163,639	Dominant
Suppressed pVL	$83,001	$216,580	Dominant
Unsuppressed pVL	$47,171	$89,676	$9,708

*Threshold for attaining 50% clinical protection for individuals with HIV.

**Figure 4:**
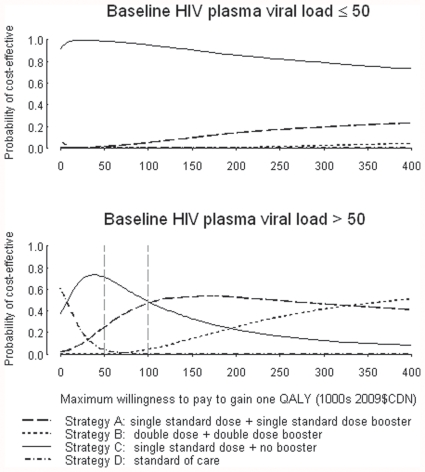
Cost-effectiveness acceptability curves.

Cost-effectiveness acceptability curves for each dosing strategy were plotted for HIV virologically suppressed and unsuppressed patients (Figure 4). Among suppressed patients, dosing strategy C (single standard dose) was the most cost-effective strategy for a wide range of threshold values of societal willingness to pay for a single QALY. Among unsuppressed patients, strategy C was the most cost-effective strategy for thresholds of up to $100,000; at a threshold of $100,000, the probabilities of Strategies A and C being the most cost-effective were nearly equal, however. Strategy A was the most cost-effective for thresholds of $100,000–$330,000 per QALY, with strategy B becoming the most cost-effective strategy at $330,000 per QALY gained.

Given the uncertainty surrounding mean ICER estimates for individuals with unsuppressed plasma viral load in particular, we calculated the EVPI of conducting further research to reduce uncertainty in the decision rule to provide strategy A (single dose+booster) versus strategy C (single dose) for this cohort. For a threshold cost-effectiveness ratio of λ = $100,000 per QALY and an estimated annual prevalence of 15,600 HIV-positive individuals with unsuppressed plasma viral load in Canada ((65,000 individuals with HIV in Canada [35])x(24% of patient within study sample with unsuppressed plasma viral load – assumed to be representative of population) = 15,600), we found that the value of further research is ($5.68 per individual * 15,600 individuals = $88,608) in the first year of implementation, and $417,973 over a 5-year timeframe.

## Discussion

Using a novel modeling approach to determine the cost-effectiveness of influenza dosing strategies incorporating individual level data on HAI titre response, we found that ensuring standard, single-dose inactivated, non-adjuvanted intramuscular influenza vaccination for all HIV/AIDS patients is a cost-effective strategy. Mean ICERs for the administration of a booster dose (strategy A: single standard dose+single standard dose booster) for unsuppressed patients were within the range of commonly accepted willingness to pay thresholds of $50,000–$100,000 [36]. While the level of uncertainty regarding the decision to implement strategy A versus C for unsuppressed patient was high, value of information analysis found that the maximum acceptable cost of reducing decision uncertainty in implementing strategy A for individuals with unsuppressed pVL was $418,000 - likely below the expense of conducting a larger-scale trial for this patient sub-group. The results presented in this manuscript therefore do not support a policy to implement this strategy on a national scale in Canada.

These conclusions are based on the best estimates of the model parameters available. One-way sensitivity analyses revealed that Strategy A (single standard dose+single standard dose booster) may be cost-effective for unsuppressed individuals at or below age 25, in years of very high ILI attack rates, and with higher case fatality rates than we used in our baseline analysis. These factors should be considered in future policy decisions regarding influenza vaccination among individuals with HIV/AIDS. Further, it is plausible that our estimate on the rate of vaccination of HIV patients in community settings is over-estimated. A report by Klein et al. [37] suggested that only 78% of persons living with HIV who presented to an HIV clinic with respiratory syndromes from 2003–2006 were vaccinated with standard single dose influenza vaccine. Decreasing the proportion of individuals vaccinated in the comparator cohort (strategy D: standard of care) would act to decrease ICERs for each of the dosing strategies assessed; rank-ordering of the alternative strategies would, however, remain constant.

Given that this cost-effectiveness analysis was conducted alongside a clinical trial, our analytic approach differs from those typically applied in cost-effectiveness studies on vaccines. Kim and Goldie [38] reported that some 90% of such studies published up to 2005 were aggregate (population-level), deterministic models. We created a closed, static and stochastic Markov cohort model, taking advantage of individual-level data on an intermediate outcome (HAI titre) used to determine the probability of clinical protection against influenza. Dynamic simulation models have the advantage of incorporating herd immunity in a population. Because our study compared interventions in which all cohorts were vaccinated and focused on a small subset of the population which is potentially more vulnerable to the influenza epidemic, we chose not to model herd immunity explicitly. We have demonstrated that our modeling approach can be applied successfully and should be considered for future cost-effectiveness analyses of experimental vaccine strategies tested with randomized controlled trial data.

A key factor in the modeling process was that the probability of ILI was estimated from an intermediate outcome (HAI titre) rather than from actual ILI cases. The total number of recorded ILI events during the period of conduct of the study upon which this analysis is based was low. As such, the trial was insufficiently powered to detect differences in ILI rates between treatment arms. As our analysis was intended to be generalizable across influenza seasons with different attack rates, we chose to use external estimates drawn from a wide time range.

Several limitations should be considered when interpreting our analysis. The variation in estimated ILI rates was large, due to the relatively small sample size of the study and heterogeneity in HAI titre response profiles. However, we feel this novel strategy is an important innovation in the study of influenza vaccine effectiveness given the limited resources available for publicly-funded clinical trials and the prevalence of use of HAI titres as outcomes in such studies. The variation in outcomes was accounted for in probabilistic sensitivity analysis, which verified the conclusions of the deterministic analysis. Finally, we have not included the potential administrative and marketing expenses that would be required to ensure total vaccine coverage among individuals with HIV/AIDS, nor the logistical requirements to execute a policy providing differential care for suppressed and unsuppressed individuals with HIV/AIDS. Not all methods of determining influenza vaccine immunogenicity were considered in our analyses [18], [39], [40]. Our analysis is based on influenza immunogenicity achieved with inactivated, non-adjuvanted vaccine administered intramuscularly. Similar economic analyses are warranted to assess increased antigen dose and/or booster dosing strategies with live, attenuated vaccines and with vaccines utilizing adjuvants. Low representation of women and those with CD4 counts below 200 cells/ml may limit applicability of our findings to these populations.
